# Supplementary material for: The short isoform of Tango1 is dispensable for zebrafish survival but is required for skeletal patterning and integrity
Source: Biol Open. 2025 Nov 27;14(11):bio062117. doi: 10.1242/bio.062117 (PMC12690545; doi:10.1242/bio.062117)
Supplement: Supplementary information [file biolopen-14-062117-s1.pdf]

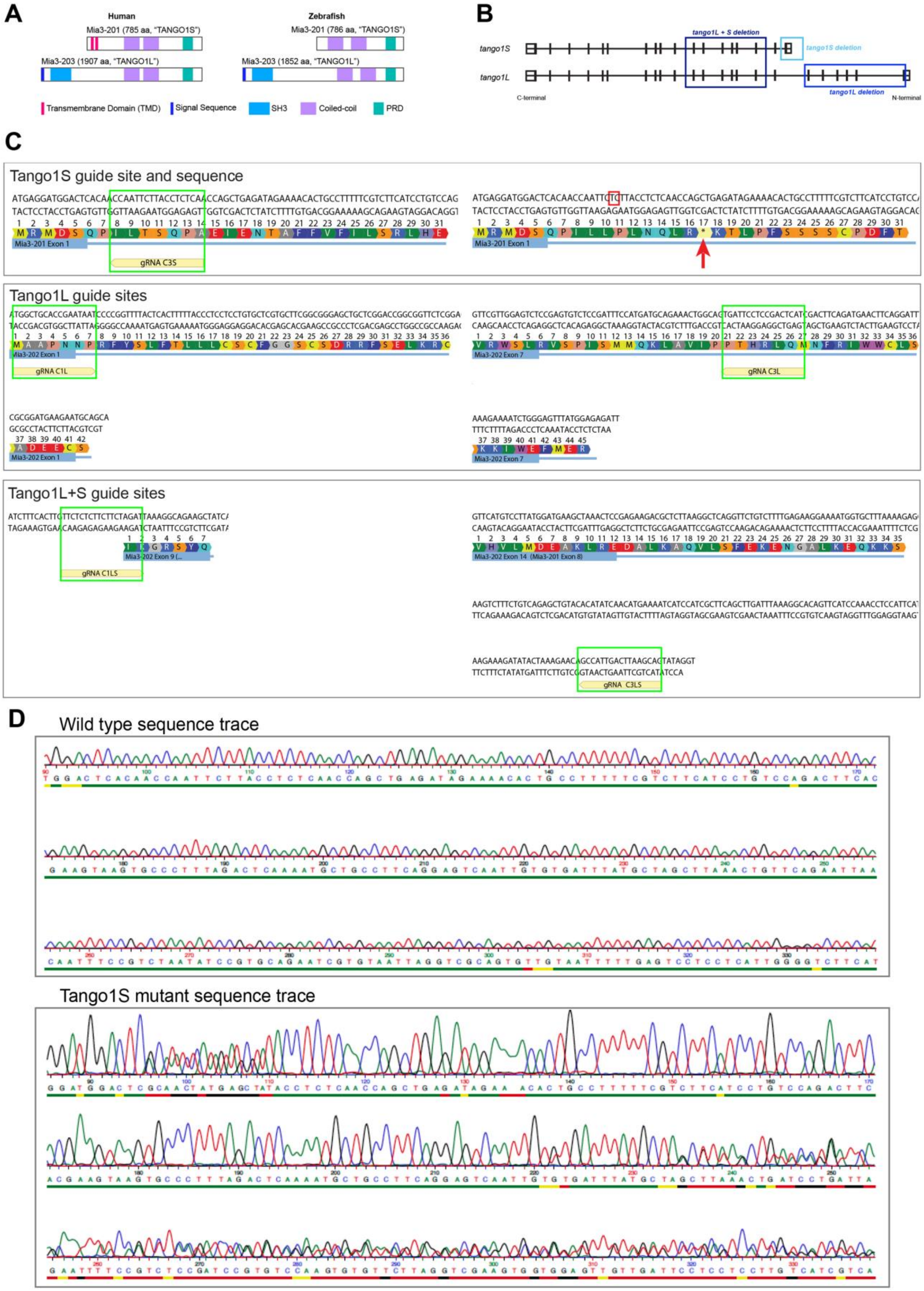

Biology Open • Supplementary information

Fig. S1. Location of guide sites for induction of mutations in the short and long isoforms of *tango1*. (A – C) Exon regions targeted by CRISPR guides to cause mutations in tango1S (A), tango1L (B), and both tango1L+S (C). Amino acid translation for the region shown by coloured letters under genomic sequence, exon targeted shown in blue box under amino acid sequence and guide sites annotated by yellow arrows, direction of arrow represents the strand the guide binds to (right facing arrow = forward strand, left facing arrow = reverse strand). For tango1S, both the wild type and mutant sequence are shown in A with the mutant having a two base pair insertion (red box) in exon 1 which introduces a premature stop codon downstream in the exon (red arrow). Tango1L and double isoform mutants have a large deletion which is visible on a DNA gel following PCR amplification of the region.

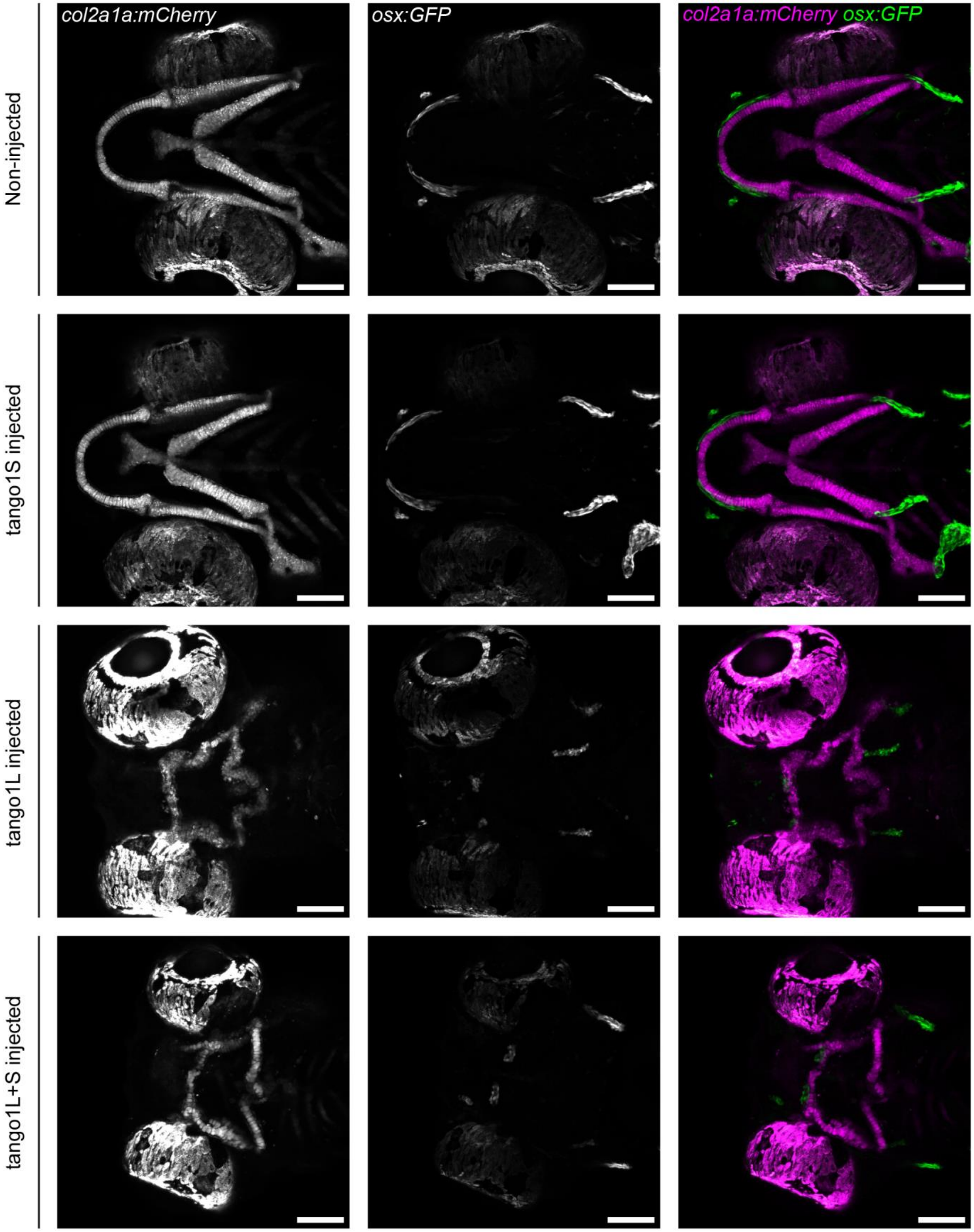
col2a1a:mCherry
osx:GFP
col2a1a:mCherry osx:GFP
Non-injected
tango1S injected
tango1L injected
tango1L+S injected

Fig. S2. Expression of *col2a1a* is unchanged in larval zebrafish lacking the tango1L isoform. Representative maximum projections of confocal image stacks taken of the lower jaw of *tg(col2a1aBAC:mCherry); (Ola.Sp7:NLS-GFP)* double transgenic fish at 5dpf. Scale bar = 250mm.

Table S1. CRISPR guides against zebrafish *tango1*

Guide	Guide sequence	Exon targeted	Protein	Sequencing primers 5’ – 3’
gRNA C3S	GCTGGTTGAGAGGTAAGAAT	Exon 1	tango1S	**FWD** AAAATCCACCGGCTTCCGAT
				**REV** AAGCCCATGTGGCACTTTCT
gRNA C1L	ATCTGAAGTCGATGAGTCGG	Exon 1	tango1L	**FWD** TGTTGGTGCTGCTATCTC
gRNA C3L	TGGCTGCACCGAATAATCCC	Exon 7		**REV** GTAGTACTCACAATCTCTCC
gRNA C1LS	TCTCTCTTCTTCTAGATTAA	Exon 9 (Mia3-202) Exon 3 (Mia3-201)	tango1L+S	**FWD** GATGTTCTTCTGGAGGACGG
gRNA C3LS	ACCTATACTGCTTAAGTCAA	After exon 14 (Mia3-202) After exon 8 (Mia3-201)		**REV** GTGGAACTGCAGGAAGAGTG

Supplementary Animal Information

Sections in this document correspond to the sections of the ARRIVE checklist for which they provide further detail.

The ARRIVE Essential 10

Study Design

For each experiment the groups being compared are a non-injected control group and 3 (in some case 4) experimental groups. The experimental groups are zebrafish injected with different guides: tango1S, tango1L and tango1L+S to target different protein isoforms with the fourth experimental group being stable tango1S mutants with the same insertion in exon1 (as opposed to crispants in the other 3 groups). The experimental unit in this study is 1 zebrafish.

Inclusion and Exclusion Criteria

No specific inclusion or exclusion criteria were applied in this study because all viable zebrafish were included. The aim of the study was to characterise an unknown phenotype, and therefore it was essential to retain all data points to capture the full range of potential variation. This approach avoided bias and ensured a comprehensive and representative analysis of the phenotype under investigation.

Randomisation

Randomisation was not used in this study. To minimise potential confounding variables in this study, we employed several measures. Injections were performed into the yolk at the same developmental stage (1-cell stage) using consistent injection volumes and concentrations, and by the same trained individual to reduce technical variability. All larvae were reared under identical environmental conditions.

Blinding

Biology Open • Supplementary information

Blinding was achieved by performing all imaging, data collection, and analysis without knowledge of the genotype. Genotypes were only determined after the completion of data analysis, ensuring that the assessment of phenotypes was unbiased.

Experimental animals

Wild-type zebrafish of the TL/Ekk strain were used for all experiments and injections. Sex is not decided in zebrafish until around 3 months of age so was not considered for larval experiments, in adult zebrafish sex was annotated and equal numbers of male and female fish used from each genetic background to avoid sex-specific skewing of the data. Weight of the zebrafish was not measured.

Zebrafish used in this study were bred in-house at the University of Bristol from adult stock maintained under standard conditions. Embryos were obtained from natural pairwise matings of the TL/Ekk line and a subset of these were injected at the 1-cell stage. Genotype was determined following phenotypic analysis; details of this process are listed in the methods section. Breeding animals were considered healthy based on normal morphology and behaviour assessed daily by experienced aquarium staff and no animals used for breeding had been subjected to any prior experimental procedures. Samples are sent from the zebrafish facility for health screens and disease testing at regular intervals to ensure the general population and system health.

The Recommended Set

Background

Zebrafish are an excellent animal model for studying genetic mutations affecting extracellular matrix (ECM) secretion due to several key biological and practical advantages. Firstly, zebrafish share a high degree of genetic and molecular conservation with humans, including many genes involved in ECM production and regulation. This homology allows insights gained from zebrafish studies to be directly relevant to understanding human biology and disease mechanisms.

Biology Open • Supplementary information

The transparency of zebrafish embryos facilitates real-time, *in vivo* visualization of ECM deposition and tissue morphogenesis, enabling detailed analysis of how specific genetic mutations disrupt ECM secretion and organisation in a 3D, whole organism context. Zebrafish develop rapidly, and their embryos are externally fertilized and accessible, allowing for efficient genetic manipulation techniques such as CRISPR/Cas9 to induce targeted mutations.

Furthermore, zebrafish possess similar ECM components to humans, including collagens, laminins, proteoglycans, and fibronectins, which form the structural scaffold essential for tissue integrity, cell signalling, and organ development. Mutations affecting ECM secretion in zebrafish often result in phenotypes such as skeletal abnormalities, craniofacial defects, and compromised tissue integrity, which parallel human connective tissue disorders like osteogenesis imperfecta, Ehlers-Danlos syndrome, and certain muscular dystrophies.

By modelling ECM-related genetic mutations in zebrafish, we can dissect the cellular pathways underlying secretion defects, study the impact on tissue architecture and function, and screen for potential therapeutic interventions. This translational relevance makes zebrafish a powerful and cost-effective model for advancing the understanding of ECM biology and its role in human health and disease.

Housing and Husbandry

Adult zebrafish were housed in autoclavable 3.5 litre tanks from Tecniplast. They were housed at a maximum density of 20 individuals per tank. To set up zebrafish crosses, breeding pairs of females and males (ratio of 2:1) were housed overnight in breeding boxes with slotted inserts. The following morning, adults were returned to their system tanks and eggs collected. Unfertilised eggs and other debris were removed, and fertilised embryos kept in 50ml petri dishes containing Danieau's solution at 28°C until 5dpf when larvae were entered into the main zebrafish facility and were grown to adulthood.

Biology Open • Supplementary information

Animal Care and Monitoring

All experimental protocols were approved by the relevant institutional ethical review boards and conducted in accordance with the Animals (Scientific Procedures) Act 1986 and associated Home Office guidelines. Measures were taken to minimise pain, suffering, and distress, including the use of anaesthesia for procedures involving protected stages of zebrafish and maintenance of animals in optimal environmental conditions. The only adverse event observed was the inability of *tango1L* and *tango1L+S* mutants to survive to adulthood. This outcome was anticipated based on previous studies. Humane endpoints were established for work involving protected stages, including regular monitoring for signs of distress, abnormal morphology, or impaired motility. Larvae displaying severe abnormalities or failure to thrive were humanely culled in accordance with approved protocols unless they were being used for the survival analysis.

Data Access

Relevant data from this publication will be uploaded to open access repositories, including key findings and line information to ZFIN.

NOTE: Please save this file locally before filling in the table, DO NOT work on the file within your internet browser as changes will not be saved. Adobe Acrobat Reader (available free here) is recommended for completion.

The ARRIVE guidelines 2.0: author checklist

The ARRIVE Essential 10

These items are the basic minimum to include in a manuscript. Without this information, readers and reviewers cannot assess the reliability of the findings.

Item		Recommendation	Section/line number, or reason for not reporting
**Study design**	1	For each experiment, provide brief details of study design including:	Supplementary Animal Information
		a. The groups being compared, including control groups. If no control group has been used, the rationale should be stated.	
		b. The experimental unit (e.g. a single animal, litter, or cage of animals).	Supplementary Animal Information
**Sample size**	2	a. Specify the exact number of experimental units allocated to each group, and the total number in each experiment. Also indicate the total number of animals used.	Figure legends
		b. Explain how the sample size was decided. Provide details of any *a priori* sample size calculation, if done.	Methods, Power Calculations
**Inclusion and exclusion criteria**	3	a. Describe any criteria used for including and excluding animals (or experimental units) during the experiment, and data points during the analysis. Specify if these criteria were established *a priori*. If no criteria were set, state this explicitly.	Supplementary Animal Information
		b. For each experimental group, report any animals, experimental units or data points not included in the analysis and explain why. If there were no exclusions, state so.	Supplementary Animal Information
		c. For each analysis, report the exact value of *n* in each experimental group.	Figure legends
**Randomisation**	4	a. State whether randomisation was used to allocate experimental units to control and treatment groups. If done, provide the method used to generate the randomisation sequence.	Supplementary Animal Information
		b. Describe the strategy used to minimise potential confounders such as the order of treatments and measurements, or animal/cage location. If confounders were not controlled, state this explicitly.	Supplementary Animal Information
**Blinding**	5	Describe who was aware of the group allocation at the different stages of the experiment (during the allocation, the conduct of the experiment, the outcome assessment, and the data analysis).	Supplementary Animal Information
**Outcome measures**	6	a. Clearly define all outcome measures assessed (e.g. cell death, molecular markers, or behavioural changes).	Results
		b. For hypothesis-testing studies, specify the primary outcome measure, i.e. the outcome measure that was used to determine the sample size.	Results
**Statistical methods**	7	a. Provide details of the statistical methods used for each analysis, including software used.	Methods, Statistics and Figure Legends
		b. Describe any methods used to assess whether the data met the assumptions of the statistical approach, and what was done if the assumptions were not met.	Methods, Statistics and Figure Legends
**Experimental animals**	8	a. Provide species-appropriate details of the animals used, including species, strain and substrain, sex, age or developmental stage, and, if relevant, weight.	Supplementary Supplementary Animal Information
		b. Provide further relevant information on the provenance of animals, health/immune status, genetic modification status, genotype, and any previous procedures.	Supplementary Supplementary Animal Information
**Experimental procedures**	9	For each experimental group, including controls, describe the procedures in enough detail to allow others to replicate them, including:	Methods
		a. What was done, how it was done and what was used.	Methods
		b. When and how often.	Methods and Supplementary Animal Information
		c. Where (including detail of any acclimatisation periods).	
		d. Why (provide rationale for procedures).	Introduction and Supplementary Animal Information
**Results**	10	For each experiment conducted, including independent replications, report:	
		a. Summary/descriptive statistics for each experimental group, with a measure of variability where applicable (e.g. mean and SD, or median and range).	Figure legends
		b. If applicable, the effect size with a confidence interval.	N/A

The Recommended Set

These items complement the Essential 10 and add important context to the study. Reporting the items in both sets represents best practice.

Item		Recommendation	Section/line number, or reason for not reporting
**Abstract**	11	Provide an accurate summary of the research objectives, animal species, strain and sex, key methods, principal findings, and study conclusions.	Abstract
**Background**	12	a. Include sufficient scientific background to understand the rationale and context for the study, and explain the experimental approach.	Introduction and Discussion
		b. Explain how the animal species and model used address the scientific objectives and, where appropriate, the relevance to human biology.	Supplementary Animal Information
**Objectives**	13	Clearly describe the research question, research objectives and, where appropriate, specific hypotheses being tested.	Abstract, Introduction and Discussion
**Ethical statement**	14	Provide the name of the ethical review committee or equivalent that has approved the use of animals in this study, and any relevant licence or protocol numbers (if applicable). If ethical approval was not sought or granted, provide a justification.	Methods
**Housing and husbandry**	15	Provide details of housing and husbandry conditions, including any environmental enrichment.	Methods and Supplementary Animal Informatio
**Animal care and monitoring**	16	a. Describe any interventions or steps taken in the experimental protocols to reduce pain, suffering and distress.	Supplementary Animal Information
		b. Report any expected or unexpected adverse events.	Supplementary Animal Information
		c. Describe the humane endpoints established for the study, the signs that were monitored and the frequency of monitoring. If the study did not have humane endpoints, state this.	Supplementary Animal Information
**Interpretation/ scientific implications**	17	a. Interpret the results, taking into account the study objectives and hypotheses, current theory and other relevant studies in the literature.	Results and Discussion
		b. Comment on the study limitations including potential sources of bias, limitations of the animal model, and imprecision associated with the results.	Results and Discussion
**Generalisability/ translation**	18	Comment on whether, and how, the findings of this study are likely to generalise to other species or experimental conditions, including any relevance to human biology (where appropriate).	Results and Discussion
**Protocol registration**	19	Provide a statement indicating whether a protocol (including the research question, key design features, and analysis plan) was prepared before the study, and if and where this protocol was registered.	N/A.
**Data access**	20	Provide a statement describing if and where study data are available.	Supplementary Animal Information
**Declaration of interests**	21	a. Declare any potential conflicts of interest, including financial and non-financial. If none exist, this should be stated.	Author's Declaration
		b. List all funding sources (including grant identifier) and the role of the funder(s) in the design, analysis and reporting of the study.	Acknowledgments
